# Nationwide Trends in Hospitalizations for Atrial Fibrillation and Flutter in the United States before and during the Outbreak of the COVID-19 Pandemic

**DOI:** 10.3390/jcm13164883

**Published:** 2024-08-19

**Authors:** Sarah Daoudi, Kevin John, Fadi Chalhoub, Jennifer Chee, Margaret Infeld, Gabby Elbaz-Greener, Munther Homoud, Jeremy N. Ruskin, E. Kevin Heist, Christopher Madias, James Udelson, Guy Rozen

**Affiliations:** 1 Cardiac Arrhythmia Center, Tufts Medical Center, Tufts University School of Medicine, Boston, MA 02111, USA; 2 Baystate Medical Center, Springfield, MA 01199, USA; 3 Department of Cardiology, Hadassah Medical Center, Faculty of Medicine, Hebrew University of Jerusalem, Jerusalem 9190400, Israel; 4 Cardiac Arrhythmia Center, Massachusetts General Hospital, Harvard Medical School, Boston, MA 02114, USA

**Keywords:** atrial fibrillation, atrial flutter, COVID-19, pandemic, arrhythmias, cardiovascular comorbidities, adverse endpoint outcomes

## Abstract

**Background/Objectives**: Atrial fibrillation (AF) and flutter (AFL) are the most common cardiac arrhythmias worldwide. Cardiovascular complications are a common manifestation of acute and post-acute COVID-19 infection. We aimed to analyze the nationwide trends in clinical characteristics and outcomes of patients hospitalized for AF/AFL before and during the COVID-19 outbreak in the U.S. **Methods**: This study is a retrospective analysis of patients, aged 18 and older, hospitalized for AF/AFL in the U.S. between 2016 and 2020. We drew data from the National Inpatient Sample (NIS) database. Baseline sociodemographic and clinical data, as well as outcomes including stroke, acute coronary syndrome (ACS), and mortality, were analyzed. Multivariable analysis was performed to identify independent associations between the different clinical and demographic characteristics and the composite endpoint of Mortality/ACS/Stroke. **Results**: An estimated total of 2,163,699 hospitalizations for AF/AFL were identified. The hospitalization volume between 2016 and 2019 was stable, averaging 465,176 a year, followed by a significant drop to 302,995 in 2020. Patients’ median age was 72 years (IQR 62–80), 50.9% were male, and 81.5% were white. The composite endpoint steadily increased from 6.5% in 2016 to 11.8% in 2020 (P_trend_ < 0.001). In a multivariable regression analysis, age > 75 (OR: 1.35; 95% CI 1.304–1.399, *p* < 0.001), ischemic heart disease (OR: 1.466; 95% CI: 1.451–1.481; *p* < 0.001), and chronic kidney disease (OR: 1.635; 95% CI: 1.616–1.653; *p* < 0.001) were associated with the composite endpoint. COVID-19 was associated with the composite endpoint outcome in the year 2020 (OR: 1.147; 95% CI: 1.037–1.265; *p* = 0.007). **Conclusions**: Hospitalization for AF/AFL dropped significantly during the first year of the COVID-19 pandemic outbreak, possibly due to patients’ avoidance of hospital visits. The composite endpoint of Mortality/ACS/Stroke uptrended significantly during the study period. COVID-19 was shown to be independently associated with the adverse composite outcome Mortality/ACS/Stroke.

## 1. Introduction

Atrial fibrillation (AF) and flutter (AFL) are the most common cardiac arrythmias globally and the most common heart rhythm abnormalities leading to hospitalization [1]. AF/AFL are a major burden on public health, with an estimated worldwide prevalence of 37.574 million cases (0.51% of the worldwide population) [2]. In addition to its significant toll on many patients’ quality of life, AF/AFL have been associated with adverse outcomes such as stroke and heart failure, consequently affecting patients’ mortality [3]. Additionally, as a result of the significant uptrend in the incidence and prevalence of AF/AFL, the cost associated with AF/AFL management is rising, driven primarily by an increase in ED visits and hospitalizations [1].

The COVID-19 pandemic outbreak at the end of 2019 overwhelmed the global healthcare system. In the months and years that followed, it has been shown that cardiovascular complications are a common manifestation of acute and post-acute COVID-19 infection due to the virus’s tendency to target the cardiovascular system [4,5]. Among other cardiovascular morbidity, cardiac arrhythmias—especially AF/AFL—have been shown to be associated with higher morbidity and mortality among patients who are hospitalized with COVID-19 infection [4,5]. These initial observations were limited by the studies’ scope and small numbers.

The purpose of this study was to examine the “real-world”, nationwide trends in AF/AFL-related hospitalizations in the United States before and during the COVID-19 era, specifically comparing trends in baseline characteristics and in-hospital outcomes between the two periods.

## 2. Materials and Methods

### 2.1. Data Sources

The data were drawn from the Nationwide Inpatient Sample (NIS), a database developed for the Healthcare Cost and Utilization Project (HCUP), sponsored by the Agency for Healthcare Research and Quality (AHRQ) [6]. The NIS is the largest collection of all-payer data on inpatient hospitalizations in the United States. The dataset represents an approximate 20% stratified sample of all inpatient discharges from U.S. hospitals. The database provides de-identified information for each hospital stay. This information includes patient-level and hospital-level factors: patients’ demographic characteristics; primary and secondary diagnoses and procedural diagnoses; AHRQ comorbidities; length of stay (LOS); hospital region; hospital teaching status; hospital bed size; and cost of hospitalization. Accurate national estimates are calculated based on this sample, using validated patient-level and hospital-level sampling weights that are provided by the NIS. We obtained data from January 2016 to December 2020 [6].

### 2.2. Study Patients and Variables

The NIS database reports diagnoses and procedures through the International Classification of Diseases-10th Revision-Clinical Modification and Procedure Coding system (ICD-10-CM/PCS). For each index hospitalization, the database provides a principal discharge diagnosis and a maximum of 39 additional diagnoses, in addition to a maximum of 25 procedures [6]. We identified patients who were 18 years of age or older who were hospitalized with a primary diagnosis of AF or AFL in the U.S. between 2016 and 2020 using the ICD-10-CM codes I48.xx (see Appendix A Table A1). Patients with missing age, sex, or mortality status (~0.07% of AF/AFL patients identified in the NIS database) were excluded from the dataset.

Utilizing the ICD-10-CM diagnosis codes (see Appendix A Table A1), we collected and analyzed baseline characteristics of patients with a primary diagnosis of AF/AFL, including common comorbidities (hypertension, acute and chronic heart failure, chronic ischemic heart disease, diabetes, chronic kidney disease, chronic respiratory disease, history of cerebrovascular disease, and COVID-19) to compare trends between the pre-COVID-19 era (2016–2019) and the COVID-19 era (2020). Patient demographic data including sex, age, race, and income percentile, as well as hospital-level data including hospital region, hospital teaching status, and hospital bed size, were also collected. For the purpose of calculating the Deyo–Charlson comorbidity index (Deyo-CCI), an additional list of comorbidities was identified from the database by using ICD-10-CM codes (detailed information on Deyo-CCI is provided in Appendix A Table A2). Deyo-CCI is a modification of the Charlson comorbidity index. It contains 17 comorbid conditions with differential weights, with a total score ranging from 0 to 33. Higher Deyo-CCI scores indicate a greater burden of comorbid diseases and are associated with mortality 1 year after admission [7,8]. The index has been used extensively in studies from administrative databases, with proven validity in predicting short- and long-term outcomes [8,9].

### 2.3. Study Outcomes

We also utilized the ICD-10 codes (see Appendix A Table A1) to identify in-hospital complications and outcomes of the study population, including stroke, acute coronary syndrome (ACS), and in-hospital mortality, to compare trends between the pre-COVID-19 and COVID-19 eras. Multivariable analysis was performed to identify predictors of a composite endpoint of Mortality/ACS/Stroke.

### 2.4. Statistical Analysis

Based on recommendations from AHRQ for analysis of survey data, variable DISCWT was used for sampling weights to account for sampling methods used in the construction of the NIS dataset [10]. Analyses of temporal trends for AF hospitalizations were conducted with the Cochrane–Armitage modification of the Chi-square test and Cuzick’s non-parametric test for trend for categorical and continuous variables, respectively [11,12,13]. Multivariable logistic regression analysis was utilized to identify clinical characteristics that were independently associated with the outcomes. In all comparisons, a *p*-value of <0.01 was considered significant. Statistical analysis was conducted using SPSS software version 25.0 (IBM Corp., Armonk, NY, USA) and R version 4.3.2 (R Foundation for Statistical Computing, Vienna, Austria). All tests were two-tailed.

## 3. Results

### 3.1. Trends in AF/AFL Hospitalizations

**Figure 1:**
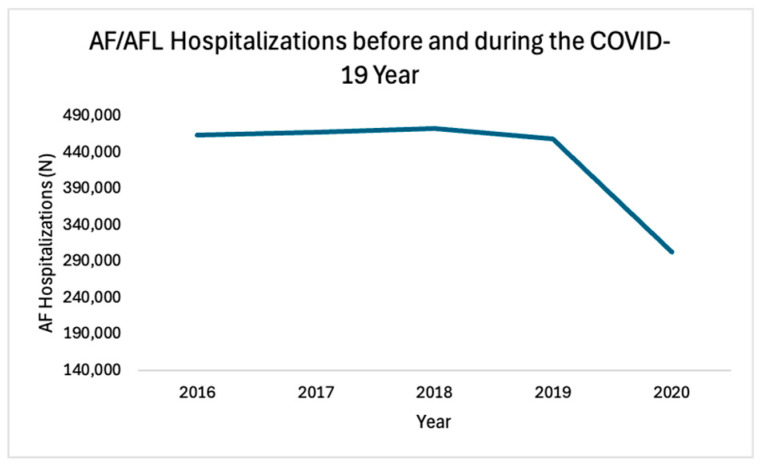
AF/AFL Hospitalizations from 2016 to 2020. From 2016 to 2019, AF/AFL hospitalization volume was stable, followed by sudden drop in hospitalizations in 2020 (COVID-19 year).

Out of the 34,955,252 U.S. hospitalizations documented in the NIS database (20% sample of all U.S. hospitalizations) between 2016 and 2020, a total of 432,740 hospitalizations with a primary discharge diagnosis of AF or AFL were included in the analysis based on the inclusion criteria. After weighting, these represent an estimated total of 2,163,699 hospitalizations for AF/AFL in the U.S. during the study period. From 2016 to 2019, the AF/AFL hospitalization volume stayed stable, with an average of 465,176 hospitalizations per year between 2016 and 2019, followed by a significant 34.9% drop in hospitalizations to 302,995 in the first year of the COVID-19 pandemic outbreak (*p* < 0.001) (Figure 1).

The demographic and clinical characteristics of the study population are presented in Table 1. The patients’ median age was 72 years (IQR 62–80), 50.9% were male, and 81.5% were white. A total of 39% of the patients were 75 years or greater in age, and 68.5% were insured by Medicare. The most common comorbidities were hypertension (79.4%), chronic ischemic heart disease (32.6%), and chronic respiratory disease (24.9%). COVID-19 infection was diagnosed in 1.2% of the study patients hospitalized in 2020.

When compared with the pre-COVID-19 era (2016–2019), patients who were admitted with AF/AFL in 2020 were statistically more likely to be black and to be male, with more chronic renal disease (*p* < 0.001) (Table 1). Hospitals in the South had the most admissions (40.9%), followed by the Midwest (24.1%), the Northeast (19.7%), and the West (15.3%). In addition, there was a significant increase in patients’ individual comorbidities, reflected by a higher average Deyo-CCI score in 2020 in comparison to the pre-COVID-19 years (2016–2019), *p* < 0.001 (Table 1).

As documented in Table 2, there was a minimal change in the rate of in-hospital mortality from 0.8% during pre-COVID-19 to 1% in the COVID-19 era (*p* = 0.007). The rate of stroke did not change significantly during the study period (*p* = 0.3816). However, a 60% increase in the rate of ACS was documented in 2020 in comparison to the pre-COVID-19 years, *p* < 0.001 (Table 2). This resulted in a 51% increase in the composite endpoint in the COVID-19 year from an average 7.8% pre-COVID-19 to 11.8% in 2020 (*p* < 0.001) (Table 2).

**Figure 2:**
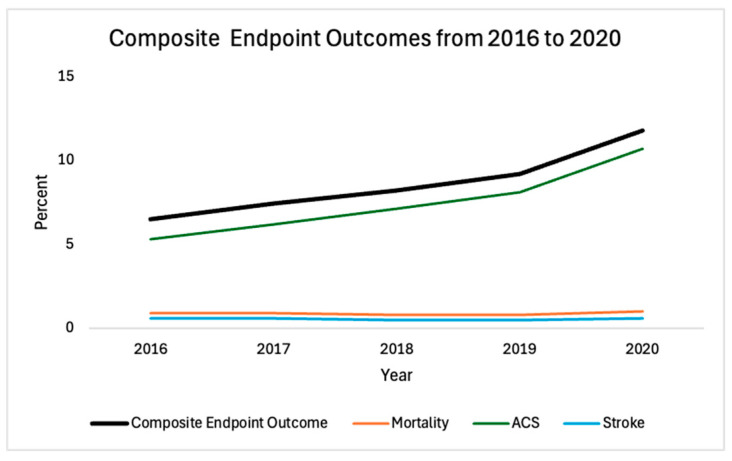
Prevalence of the composite endpoint Mortality/ACS/Stroke from 2016 to 2020. The incidence of acute coronary syndrome, as well as the composite adverse endpoint outcome Mortality/ACS/Stroke, increased steadily from 2016 to 2019, with a noticeable increase in 2020.

The composite endpoint of Mortality/ACS/Stroke steadily increased from 2016 through 2019, with a spike in 2020, driven by the increase in ACS incidence (Figure 2).

**Figure 3:**
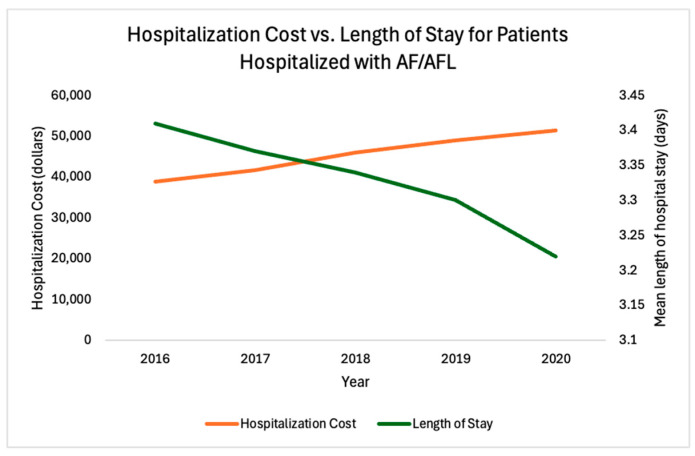
Hospitalization costs vs. length of hospital stay from 2016 to 2020. The mean length of a hospital stay steadily decreased from 2016 to 2020, while the hospitalization cost steadily increased from 2016 to 2020.

In addition, as shown in Figure 3, there was a steady, approximately linear decrease in the mean length of hospital stay for patients hospitalized with AF from 2016 to 2020. Nevertheless, the mean hospitalization cost for these patients followed an opposite trend, increasing steadily from 2016 to 2020.

### 3.2. Trends in Adverse Endpoint Outcomes

Even in the years before the COVID-19 pandemic, the data showed an increased incidence of the composite endpoint outcome Mortality/ACS/Stroke. A univariable analysis reveals that all the following comorbidities were correlated with increased rates of the composite endpoint outcome in the years 2016–2020: hypertension (OR: 1.358; 95% CI: 1.341–1.376; *p* < 0.001), acute heart failure (OR: 1.636; 95% CI: 1.621–1.657; *p* < 0.001), chronic heart failure (OR: 1.189; 95% CI: 1.176–1.203; *p* < 0.001), ischemic heart disease (OR: 1.800; 95% CI: 1.782–1.817; *p* < 0.001), diabetes mellitus (OR: 1.051; 95% CI: 1.038–1.063; *p* < 0.001), chronic kidney disease (OR: 2.426; 95% CI: 2.401–2.451; *p* < 0.001), chronic respiratory disease (OR: 1.103; 95% CI: 1.091–1.115; *p* < 0.001), and a history of cerebrovascular disease (OR: 1.161; 95% CI: 1.144–1.178; *p* < 0.001).

### 3.3. Inpatient Catheter Ablation Procedure and Outcomes

An additional analysis of AF/AFL hospitalizations revealed that approximately 0.8% of patients (n = 17,910) who were hospitalized for AF/AFL from 2016 to 2020 also underwent a catheter ablation procedure to treat their AF/AFL during the same index hospitalization. Of these patients, 65.3% were male, 81.5% were white, and the median age was 67 years (IQR 59–74). The most common comorbidities were also hypertension (74.5%), chronic ischemic heart disease (33.5%), and chronic respiratory disease (23.2%). COVID-19 infection was not diagnosed in any of these patients. When compared to individuals who did not undergo a catheter ablation procedure, these patients were statistically more likely to be under the age of 75 and less likely to have hypertension (*p* < 0.001), acute heart failure (*p* < 0.001), or a history of cerebrovascular disease (*p* < 0.001). These patients were also more likely to have been admitted to a large, urban hospital (*p* < 0.001).

Regarding outcomes, individuals who underwent a catheter ablation procedure during their AF/AFL hospital admission had a lower likelihood of developing the adverse endpoint outcome Mortality/ACS/Stroke (*p* < 0.001). These individuals also had a longer average length of stay (*p* < 0.001).

### 3.4. Multivariable Regression Analysis for Predictors of Endpoint Outcomes

**Figure 4:**
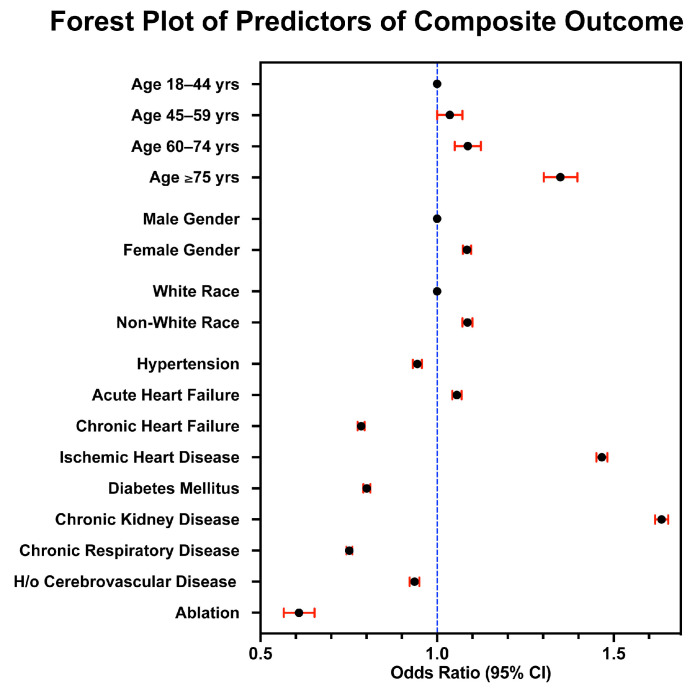
Forest Plot predictors of the composite adverse outcome (Mortality/ACS/Stroke). Non-white race, female gender, age > 60, acute heart failure, ischemic heart disease, and chronic kidney disease were independently associated with the composite endpoint outcome. Hypertension, chronic heart failure, diabetes mellitus, chronic respiratory disease, and a history of cerebrovascular disease were associated with a lower risk of the composite endpoint outcome.

In a multivariable regression analysis for the years 2016 to 2020, female gender (OR: 1.085; 95% CI: 1.073–1.096; *p* < 0.001), non-white race (OR: 1.086; 95% CI: 1.072–1.100; *p* < 0.001), acute heart failure (OR: 1.056; 95% CI: 1.043–1.069; *p* < 0.001), ischemic heart disease (OR: 1.467; 95% CI: 1.451–1.481; *p* < 0.001), and chronic kidney disease (OR: 1.635; 95% CI: 1.617–1.654; *p* < 0.001) were shown to be independently associated with the composite endpoint outcome of Mortality/ACS/Stroke (Figure 4). In addition, “catheter ablation” (OR: 0.608; 95% CI: 0.566–0.653; *p* < 0.001) was found to be negatively associated with the composite endpoint outcome of Mortality/ACS/Stroke (Figure 4).

Interestingly, in a multivariable analysis, hypertension (OR: 0.944; 95% CI: 0.931–0.957; *p* < 0.001), chronic heart failure (OR: 0.785; 95% CI: 0.775–0.795; *p* < 0.001), diabetes mellitus (OR: 0.801; 95% CI: 0.791–0.811; *p* < 0.001), chronic respiratory disease (OR: 0.752; 95% CI: 0.743–0.760; *p* < 0.001), and a history of cerebrovascular disease (OR: 0.936; 95% CI: 0.922–0.951; *p* < 0.001) were found to be associated with a lower risk of the composite endpoint outcome of Mortality/ACS/Stroke (Figure 4).

An analysis of 2020 hospitalizations also showed COVID-19 infection to be independently associated with the composite endpoint outcome of Mortality/ACS/Stroke (OR: 1.147; 95% CI: 1.037–1.265; *p* = 0.007).

## 4. Discussion

This study reports trends in hospitalizations for atrial fibrillation and flutter in the United States over a 4-year period from 2016 to 2020. The main findings of our analysis are as follows: (1) the AF/AFL hospitalization volume was steady during the pre-COVID-19 years from 2016 to 2019, followed by sharp decline in 2020, corresponding with the onset of the COVID-19 pandemic; (2) the patient population admitted for AF/AFL in the COVID-19 era included more male and black patients, with a higher prevalence of chronic renal failure; (3) there was a significant spike in the composite endpoint of Mortality/ACS/Stroke during the COVID-19 outbreak, as well as slightly higher in-hospital mortality in 2020 compared to the pre-COVID-19 era; and (4) COVID-19 was independently associated with a significantly higher rate of the adverse composite endpoint outcome in the year 2020.

The data in this study show a slight increase in hospital admissions for AF/AFL from 2016 to 2019, although not reaching statistical significance in this analysis. The existing literature shows an increasing trend in AF hospitalizations over the years [1,14,15,16,17,18]. However, we were unable to find studies looking into the trends for recent years (2016–2020), and it is possible that the uptrend in AF admissions was attenuated during these years, as shown in our analysis. This is to our knowledge the first nationwide analysis of trends in hospitalized AF/AFL population characteristics during the outbreak of the COVID-19 pandemic, showing a sharp decline in hospitalizations and increase in comorbidities of hospitalized AF/AFL patients in the COVID-19 era. These findings align with the CDC report published in June 2020, estimating that 41% of U.S. adults delayed or avoided medical care due to COVID-19 concerns [19]. A literature review reveals that other cardiac conditions were undertreated during the COVID-19 pandemic. Mafham et al. reported that hospital admissions for acute coronary syndrome in England declined between February and March 2020 [20]. Similarly, a Beijing inpatient database study found that admissions for STEMI, Non-STEMI, and unstable angina all declined significantly between January and June 2020 [21].

In our study, men made up the majority of AF/AFL hospitalizations compared to women (50.9% vs. 49.1%), which is consistent with the existing literature. Prior studies report that the age-standardized prevalence of AF/AFL has been consistently higher in men than in women [22,23]. A majority of hospital admissions (40.9%) for AF/AFL in our study occurred in southern states of the U.S. (Table 1). The southern states are well known for an increased prevalence of known risk factors for AF/AFL like obesity, diabetes mellitus, hypertension, and heart failure, as well as incidence of stroke, compared with other regions [24,25].

Individuals who underwent a catheter ablation procedure during their hospitalization showed lower rates of the adverse composite endpoint outcome in comparison to patients who did not undergo catheter ablation. The multivariable analysis also revealed catheter ablation to be negatively associated with the adverse composite endpoint outcome for patients hospitalized with AF/AFL. These findings are in alignment with the growing evidence suggesting that rhythm control therapies are favorable rate control therapies in patients with AF [26]. Given that the percentage of patients who underwent an ablation procedure in the study was very limited (<1%), further studies, with longer follow-up, are needed to assess the effect of inpatient ablation procedures on patients admitted to hospitals with AF/AFL.

Interestingly, some comorbidities like hypertension, chronic heart failure, diabetes mellitus, chronic respiratory disease, and a history of cerebrovascular disease were shown to be protective of the adverse composite outcome in a multivariable analysis. The nature of the NIS database limits our ability to dive deeper into analyzing the nature of this protective effect. One possible explanation, for instance, is that some of these comorbidities are associated with obesity—which has a well-documented protective effect in patients with various cardiovascular conditions [27,28]. It is also likely that patients in our study who had pre-existing comorbid conditions prior to their AF hospitalizations captured in this study were more likely to have been followed closely by their primary care physician and more likely to have been treated with anticoagulation medications and/or beta blockers prior to hospitalization. It is well known that AF is often asymptomatic and can go undetected in about 30% of patients [29,30]. This can result in a greater chance of adverse outcomes for these AF patients following hospitalization. Thus, we postulate that the protective effects of hypertension, chronic heart failure, diabetes mellitus, chronic respiratory disease, and a history of cerebrovascular disease are a result of early diagnosis and better follow-up for patients with a greater number of comorbid conditions to AF.

Our nationwide analysis also showed a statistically significant change in the rate of in-hospital mortality in 2020 compared to the pre-COVID-19 era. A study by Coromilas et al. showed that 70% of patients who were hospitalized with COVID-19 who developed a cardiac arrhythmia developed atrial arrhythmias, and that the presence of arrhythmia was associated with significant morbidity and mortality [31]. In alignment with these findings, our study’s multivariable analysis for the year 2020 also showed COVID-19 to worsen the composite adverse outcome endpoint in patients admitted for AF/AFL. Similar findings were reported by Alharbi et al. showing that COVID-19 infection is worsening the outcomes of patients who are admitted for heart failure in the U.S. [32].

The mean length of hospital stay trended down over the study duration, while the total hospitalization cost trended up (Figure 3). The increase in the cost of hospital stay per day over the years, as well as the increase in cardiovascular comorbidities and associated treatments, may provide explanations for the increase in the hospitalization costs (Table 1). Medicare was the primary source of payment for patients, further reflecting an increase in hospitalizations in elderly patients. To reduce this economic burden, certain interventions, such as the implementation of “AF pathway” protocols for the emergency departments (EDs), utilizing ED observation units versus hospital admission, and using low-molecular-weight heparin and direct oral anticoagulants versus unfractionated heparin, may be employed [33,34]. These methods could limit the number of hospitalizations and decrease the length of stay and have previously been described as potential means to reduce the cost associated with the treatment of AF/AFL [33,34].

Several limitations of this study need to be acknowledged. First, the NIS database is a retrospective administrative database that contains discharge-level records and, as such, is susceptible to coding errors. Secondly, the lack of patient identifiers in the NIS database prevented us from using other outcome variables and mortality measures, such as at-30-days mortality. We could only capture events that occurred in the same index of hospitalization. The NIS database also does not include detailed information about patients’ clinical characteristics, including the duration of AF, medication, blood tests, etc. Therefore, we cannot rule out residual confounding within the associations we observed.

## 5. Conclusions

The AF/AFL hospitalization volume in the U.S. remained stable between 2016 and 2019, followed by a significant decline in 2020, the year of the COVID-19 pandemic’s outbreak, possibly due to patients’ attempts to avoid hospital visits. The composite endpoint of Mortality/ACS/Stroke, as well as hospitalization costs, uptrended significantly during the study period, in parallel to the uptrend in patients’ comorbidities. COVID-19 was found to be independently associated with the adverse composite endpoint outcome in the year 2020.

## Figures and Tables

**Table 1:** Baseline characteristics of patients hospitalized with atrial fibrillation or flutter before (2016–2019) and during (2020) the COVID-19 era.

		Total	2016–2019 (Mean)	2020
N	Unweighted	432,740	93,035	60,599
	Weighted	2,163,699	465,176	302,995
Age (%)	18–44	4	4	4.4
	45–59	17.1	17.1	16.9
	60–74	40	39.9	40.5
	75 or older	38.9	39	38.2
Gender (%)	Male	50.9	50.8	51.5
	Female	49.1	49.2	48.5
Race (%)	White	81.5	81.6	81
	Black	8.6	8.5	9.2
	Hispanic	5.9	6	5.7
	Asian/Pacific Islander	1.5	1.5	1.5
	Native American	0.4	0.4	0.4
	Others	2.1	2.1	2.1
Comorbidities (%)	Hypertension	79.4	79.2	80.3
	Chronic Heart Failure	21.2	21.1	21.9
	Ischemic Heart Disease	32.6	32.5	33
	Diabetes Mellitus	21.1	21.3	20.3
	Chronic Kidney Disease	20.1	19.6	23.5
	Chronic Respiratory Disease	24.9	25	24.4
	H/O Cerebrovascular Disease	11.5	11.5	11.7
	COVID-19	0.2	0	1.2
Deyo-CCI, %	0	22.6	22.8	21.4
	1	25.6	25.7	24.9
	2 or higher	51.8	51.4	53.7
Disposition of Patients (%)	Routine	74.6	74.6	74.6
	Transfer to Short- term Hosp	2.1	2.1	2
	Transfer other	10.1	10.4	8.7
	Home Healthcare	12	11.8	13.1
	Against Medical Advice	1.2	1.1	1.5
Status of hospital (%)	Rural	10.6	10.6	10.7
	Urban non-teaching	23.1	23.6	19.8
	Urban teaching	66.3	65.8	69.5
Hospital Bed Size (%)	Small	21.1	20.7	23.6
	Medium	30.1	30.2	29.4
	Large	48.8	49.1	46.9
Primary Expected Payer (%)	Medicare	68.5	68.6	67.3
	Medicaid	6.3	6.2	6.9
	Private	20.5	20.5	20.5
	Self-pay	2.5	2.4	2.7
	No change	0.2	0.2	0.2
	Other	2.1	2	2.4
Region of hospital (%)	Northeast	19.7	19.8	19
	Midwest	24.1	24.2	23.2
	South	40.9	40.7	42.2
	West	15.3	15.3	15.5

**Table 2:** Outcomes of patients hospitalized with AF/AFL in the U.S. before (2016–2019) and during (2020) the COVID-19 era.

	Total	2016–2019	2020	Percent Change	*p*-Value
Overall mortality (%) *	0.9	0.8	1	25%	0.007
Acute Heart Failure	18.6	18.6	18.9	1.61%	0.0347
Acute stroke (%)	0.6	0.6	0.6	0%	0.3816
Acute Coronary Syndrome (%) ^	7.2	6.7	10.7	60%	<0.001
Mortality/ACS/Stroke (%)	8.4	7.8	11.8	51%	<0.001
Mean length of hospital stay, days	3.34	3.35	3.22	−3.88%	<0.001
Mean hospitalization cost, USD	44,908	43,860	51,347	17%	<0.001

* Overall mortality refers to in-hospital mortality only. ^ Acute coronary syndrome refers to acute myocardial infarction, as well as other acute ischemic heart disease.

## Data Availability

The original data presented in this study are openly available in the HCUP Nationwide Inpatient Sample (NIS) database at https://hcup-us.ahrq.gov/db/nation/nis/NIS_Introduction_2020.jsp (accessed on 23 December 2022).

## Appendix A. ICD-10 CM Codes Used in the Research

**Table A1:** ICD-10 CM diagnosis and procedure codes.

Condition	ICD-10 CM Codes
AF/AFL	I48.0, I48.11, I48.19, I48.20, I48.21, I48.3, I48.4. I48.91, I48.92
Hypertension	I10, I11.0, I11.9, I12.0, I12.9, I13.0, I13.10, I13.11, I13.2 I15.0, I15.1, I15.2, I15.8, I15.9, I16.0, I16.1, I16.9
Chronic Heart Failure	I50.1, I50.20, I50.22, I50.30, I50.32, I50.40, I50.42, I50.810, I50.812, I50.814, I50.82, I50.83, I50.84, I50.89, I50.9
Acute Heart Failure	I50.21, I50.23, I50.31, I50.33, I50.41, I50.43, I50.811, I50.813
Chronic Ischemic Heart Disease	I25.1xx, I25.4x, I25.5, I25.7xx, I25.8xx, I25.9
Diabetes Mellitus	E08.42, E08.649, E08.65, E08.9, E09.22, E09.649, E09.65, E09.8, E09.9, E10.0x, E10.1x, E10.6x, E10.8x, E10.9x, E11.0x, E11.1x, E11.6x, E11.8x, E11.9x, E13.0x, E13.1x, E13.6x, E13.8x, E13.9x
Chronic Kidney Disease	N17.0, N17.1, N17.2, N17.8, N17.9, N18.1, N18.2, N18.3, N18.30, N18.31, N18.32, N18.4, N18.5, N18.6, N18.9, N19
Chronic Respiratory Disease	J40, J41.0, J41.1, J41.8, J42, J43.0, J43.1, J43.2, J43.8, J43.9, J44.0, J44.1, J44.9, J45.20, J45.21, J45.30, J45.40, J45.41, J45.42, J45.50, J45.51, J45.901, J45.902, J45.909, J45.990, J47.0, J47.1, J47.9
History of Cerebrovascular Disease	I69.0x, I69.1x, I69.2x, I69.3x, I69.4x, I69.6x, I69.7x, I69.8x, I69.9x, Z86.73
COVID-19	U07.1, J12.82
Acute Coronary Syndrome	I20.0, I20.1, I21.01, I21.02, I21.09, I21.11, I21.19 I21.21, I21.29, I21.3, I21.4, I21.9, I21.A1, I21.A9, I21.B, I22.0, I22.1, I22.2, I22.8, I22.9, I23.0, I24.0, I24.8, I24.9
Stroke	I63.0x, I63.1x, I63.2x, I63.3x, I63.40, I63.41x, I63.42x, I63.43x, I63.44x, I63.49, I63.5x, I63.8x, I63.9, I69.05x
Catheter ablation	025S0ZZ, 025S3ZZ, 025S4ZZ, 025T0ZZ, 025T3ZZ, 025T4ZZ, 02560ZZ, 02563ZZ, 02564ZZ, 02570ZK, 02570ZZ, 02573ZK, 02573ZZ, 02574ZK, 02574ZZ

**Table A2:** ICD-10 CM codes for Deyo-CCI score [27].

Condition	ICD-10 CM Codes for Deyo-CCI Score	Score
Myocardial infarction	I21.x, I22.x, I25.2	1
Congestive heart failure	I09.9, I11.0, I13.0, I13.2, I25.5, I42.0, I42.5–I42.9, I43.x, I50.x, P29.0	1
Peripheral vascular disease	I70.x, I71.x, I73.1, I73.8, I73.9, I77.1, I79.0, I79.2, K55.1, K55.8, K55.9, Z95.8, Z95.9	1
Cerebrovascular disease	G45.x, G46.x, H34.0, I60.x–I69.x	1
Dementia	F00.x–F03.x, F05.1, G30.x, G31.1	1
Chronic pulmonary disease	I27.8, I27.9, J40.x–J47.x, J60.x–J67.x, J68.4, J70.1, J70.3	1
Rheumatologic disease	M05.x, M06.x, M31.5, M32.x–M34.x, M35.1, M35.3, M36.0	1
Peptic ulcer disease	K25.x–K28.x	1
Mild liver disease	B18.x, K70.0–K70.3, K70.9, K71.3–K71.5, K71.7, K73.x, K74.x, K76.0, K76.2–K76.4, K76.8, K76.9, Z94.4	1
Diabetes	E10.0, E10.l, E10.6, E10.8, E10.9, E11.0, E11.1, E11.6, E11.8, E11.9, E12.0, E12.1, E12.6, E12.8, E12.9, E13.0, E13.1, E13.6, E13.8, E13.9, E14.0, E14.1, E14.6, E14.8, E14.9	1
Diabetes with chronic complications	E10.2–E10.5, E10.7, E11.2–E11.5, E11.7, E12.2–E12.5, E12.7, E13.2–E13.5, E13.7, E14.2–E14.5, E14.7	2
Hemiplegia or paraplegia	G04.1, G11.4, G80.1, G80.2, G81.x, G82.x, G83.0–G83.4, G83.9	2
Renal disease	I12.0, I13.1, N03.2–N03.7, N05.2–N05.7, N18.x, N19.x, N25.0, Z49.0–Z49.2, Z94.0, Z99.2	2
Any malignancy including leukemia and lymphoma	C00.x–C26.x, C30.x–C34.x, C37.x–C41.x, C43.x, C45.x–C58.x, C60.x–C76.x, C81.x–C85.x, C88.x, C90.x–C97.x	2
Moderate or severe liver disease	I85.0, I85.9, I86.4, I98.2, K70.4, K71.1, K72.1, K72.9, K76.5, K76.6, K76.7	3
Metastatic solid tumor	C77.x–C80.x	6
Acquired immunodeficiency syndrome (AIDS)	B20.x–B22.x, B24.x	6

## Appendix B. Trends in Baseline Characteristics of Patients Hospitalized with Atrial Fibrillation in the U.S. between 2016 and 2020

		**Total**	**2016**	**2017**	**2018**	**2019**	**2020**	**P (Trend)**
N	Unweighted	432,740	92,760	93,400	94,367	91,614	60,599	0.2301
	Weighted	2,163,699	463,800	467,000	471,835	458,070	302,995	
Age (%)	18–44	4	4.2	4	3.9	3.9	4.4	<0.001
	45–59	17.1	17.8	17.4	17	16.3	16.9	
	60–74	40	39	39.9	40.4	40.3	40.5	
	75 or older	38.9	39	38.8	38.8	39.5	38.2	
Gender (%)	Male	50.9	50.4	50.4	50.9	51.4	51.5	<0.001
	Female	49.1	49.6	49.6	49.1	48.6	48.5	
Race (%)	White	81.5	81.8	81.6	81.2	81.8	81	0.024
	Black	8.6	8.5	8.4	8.4	8.6	9.2	
	Hispanic	5.9	5.8	6	6.3	5.7	5.7	
	Asian/Pacific Islander	1.5	1.5	1.5	1.6	1.5	1.5	
	Native American	0.4	0.3	0.4	0.4	0.4	0.4	
	Others	2.1	2.1	2	2.1	2.1	2.1	
Comorbidities (%)	Hypertension	79.4	77.5	79	80	80.5	80.3	<0.001
	Chronic Heart Failure	21.2	19.6	20.5	21.8	22.6	21.9	<0.001
	Ischemic Heart Disease	32.6	32.3	32	32.7	33.1	33	<0.001
	Diabetes Mellitus	21.1	24	20.8	20.3	20.1	20.3	<0.001
	Chronic Kidney Disease	20.1	18.9	19.1	19.7	20.7	23.5	<0.001
	Chronic Respiratory Disease	24.9	24.8	25.1	24.9	25.1	24.4	0.237
	H/O Cerebrovascular Disease	11.5	11	11.3	11.6	11.9	11.7	<0.001
	COVID-19	0.2	0	0	0	0	1.2	<0.001
Disposition of Patients (%)	Routine	74.6	74.1	74.2	74.9	75	74.6	0.21
	Transfer to Short term Hosp	2.1	2.2	2.1	2	2.1	2	
	Transfer other	10.1	10.7	10.6	10.2	9.9	8.7	
	Home Healthcare	12	11.9	11.8	11.8	11.9	13.1	
	Against Medical Advice	1.2	1.1	1.2	1.1	1.1	1.5	
Deyo-CCI, %	0	22.6	24.2	23.4	22.2	21.6	21.4	<0.001
	1	25.6	26	26.1	25.6	25.2	24.9	
	2 or higher	51.8	49.8	50.5	52.2	53.2	53.7	
Status of hospital (%)	Rural	10.6	10.9	10.8	10.5	10.2	10.7	<0.001
	Urban non-teaching	23.1	28.5	24.1	22.2	19.6	19.8	
	Urban teaching	66.3	60.6	65.1	67.3	70.1	69.5	
Hospital Bed Size (%)	Small	21.1	19.1	20.3	21.3	22.2	23.6	<0.001
	Medium	30.1	29.7	30.8	30	30.2	29.4	
	Large	48.8	51.1	48.9	48.7	47.6	46.9	
Primary Expected Payer (%)	Medicare	68.5	68	68.7	68.9	68.9	67.3	0.00548
	Medicaid	6.3	6.2	6.3	6.2	6.2	6.9	
	Private	20.5	21.2	20.5	20.4	19.9	20.5	
	Self-pay	2.5	2.3	2.3	2.5	2.6	2.7	
	No change	0.2	0.2	0.2	0.2	0.2	0.2	
	Other	2.1	2.1	1.9	1.9	2.1	2.4	
Region of hospital (%)	Northeast	19.7	20.5	20.1	19.3	19.3	19	<0.001
	Midwest	24.1	24.2	24.3	24.3	24.1	23.2	
	South	40.9	40.3	40.4	40.9	41.1	42.2	
	West	15.3	15	15.3	15.4	15.5	15.5	

## Appendix C. Trends in Outcomes of Patients Hospitalized with AF/AFL in the U.S. between 2016 and 2020

	**Total**	**2016**	**2017**	**2018**	**2019**	**2020**	***p*-Value**
Overall mortality (%) *	0.9	0.9	0.9	0.8	0.8	1	0.644
Acute Heart Failure (%)	18.6	18.2	18.1	19.2	18.9	18.9	<0.001
Acute stroke (%)	0.6	0.6	0.6	0.5	0.5	0.6	0.175
Acute Coronary Syndrome (%) ^	7.2	5.3	6.2	7.1	8.1	10.7	<0.001
Mortality/ACS/Stroke (%)	8.4	6.5	7.4	8.2	9.2	11.8	<0.001
Mean length of hospital stay, days	3.34	3.41	3.37	3.34	3.3	3.22	<0.001
Mean hospitalization cost, USD	44,908	38,841	41,620	46,067	48,913	51,347	<0.001
	* Overall mortality refers to in-hospital mortality only. ^ Acute coronary syndrome refers to acute myocardial infarction, as well as other acute ischemic heart disease.						
